# Developing inclusive education in Portugal: Evidence and challenges

**DOI:** 10.1007/s11125-020-09504-y

**Published:** 2020-10-19

**Authors:** Ines Alves, Paula Campos Pinto, Teresa Janela Pinto

**Affiliations:** 1 University of Glasgow, 11 Eldon St, Glasgow, G3 6NH United Kingdom; 2 School for Social and Political Sciences/University of Lisbon (ISCSP/ULisboa), Lisbon, Portugal

**Keywords:** Inclusive education, Educational policy, Portugal

## Abstract

This article assesses evidence of and challenges to the development of inclusive education in Portugal, which is built on three pillars: access to, participation in, and achievement in education for all children and young people. It presents an overview of the present policy framework, followed by an analysis of available statistical data on Portuguese students with disabilities in mainstream schools. The article also discusses significant achievements at the policy and practice levels, namely the attempt to align curriculum and pedagogy and the presence of almost 100% of students with disabilities in mainstream schools. It also considers challenges, such as the issue of monitoring achievement (both at the student and system level) and investments in the system and in teacher education.

In this article, we assess evidence of and challenges to the development of inclusive education in Portugal. Inclusive education has developed from a single-layered concept, focused on “mainstreaming” students with disabilities or “special needs” into regular schools (UNESCO 1994), to a multi-layered concept which implies developing equitable quality education systems by removing barriers to the “presence, participation and achievement of all students in education” (Ainscow 2005, p. 119). Presently, the United Nations defines inclusive education as “access to and progress in high-quality education without discrimination” (UN 2016, p. 3), which requires “a process of systemic reform…to provide all students of the relevant age range with an equitable and participatory learning experience and environment that best corresponds to their requirements and preferences” (UN 2016, p. 4).

In this article, we consider the three pillars of inclusive education: access to, participation in, and achievement in education for all children and young people (UNESCO 2017, p. 13). The first concept is *access*. Moving beyond the notion of presence proposed by Ainscow (2005), which provided a first indicator of the level of “mainstreaming” in a specific context, access includes physical access but recognizes other potential sensory, intellectual, economic, and attitudinal barriers to education. For example, students with Portuguese as an additional language might be in a mainstream classroom, but if they have recently arrived in the country with little fluency in Portuguese, they might struggle to access education due to language or attitudinal barriers. *Participation* is the second key concept; it can relate to a student’s frequency of attendance but also to the student’s subjective perceptions of involvement and active engagement (Maxwell et al. 2012; Granlund 2013). The third pillar of inclusive education is *achievement*, which must transcend traditional notions of academic achievement (grades in literacy and numeracy). In this sense, it should not only measure “learner performance and achievement on standardised national or international tests” (EASNIE 2017, p. 19) but also encompass the development of a “deeper understanding of the world” and of knowledge that persists after the completion of the schooling years, such as “critical thinking, collaborative skills, creativity, independence and problem-solving ability” (EASNIE 2017, p. 19). Notions of achievement are thus related to the curriculum, and to what types and forms of knowledge are valued.

As proposed by Lingard (Lingard 2007; Lingard and Mills 2007), curriculum does not function in isolation: it is part of the “message systems of schooling”, along with pedagogy and assessment. The alignment of message systems might be easier at some levels than others. However, if these message systems send competing or conflicting messages to practitioners, it which makes it difficult to develop more inclusive education systems. For example, if schools are encouraged to be inclusive, but there is a prescriptive curriculum that does not allow teachers to adapt contents, pedagogical approaches, or assessment to different student characteristics and needs, then the resulting paradox can prevent genuine inclusiveness.

In Portugal, particularly since the last education reform in 2018, there has been a push to develop inclusive schools “where each and every student, regardless of their personal and social situation, finds responses to their potential, expectations, and needs, and develops a level of education that creates full participation, a sense of belonging, and equity, contributing to social inclusion and cohesion” (Ministério da Educação 2018). Encouragingly, Portuguese policies make a strong attempt to create an inclusive education system, an effort which seems to be accepted by most stakeholders in terms of values. However, the challenges of implementation, especially a perceived lack of resources and the concern that sharing scarce resources amongst a larger group of students might disadvantage those who are the most vulnerable (e.g., disabled students with complex needs), creates an inextricable challenge for current Portuguese inclusive education policy and practice. In our article, we aim to examine this paradox. First, we discuss the key aspects of the present education policy framework. Then, drawing from available statistical data and media representations, we provide an overview of the main outcomes and debates around the development of inclusive education in Portugal. We conclude by revisiting the three pillars of inclusive education, to discuss the achievements and the challenges that remain in implementing inclusive education in Portugal.

## The legal context in Portugal

Portugal has been internationally recognised for its progressive legal framework in the field of inclusive education (All Means All 2018). When Decree-Law 3/2008 was introduced over a decade ago, special schools started to close down. Many were transformed into “Resource Centres for Inclusion” and tasked with supporting their former students, who were placed in mainstream regular schools. More recently, following the ratification in Portugal of the Convention on the Rights of Persons with Disabilities, this pathway was furthered with the Inclusive Education Act of 2018, Decree-Law 54/2018, which set up a new regime of inclusive education (see Alves 2019).

The Inclusive Education Act (Decree-Law 54/2018) advances a pedagogical model based on the notion that all students have learning potential, as long as they receive adequate support. Thus, the methodological options underlying this decree-law are based on universal design for learning and a multilevel approach to access the curriculum. The tiered multilevel approach encompasses the implementation of three types of measures, identified in the legislation as: *universal measures*, targeted to all students in order “to promote participation and improved learning” (Decree-Law 54/2018, Art. 8); *selective measures*, aimed to fill the need for learning supports not addressed by universal measures; and *additional measures*, set in place “to respond to intense and persistent communication, interaction, cognitive or learning difficulties that require specialised resources of support to learning and inclusion” (Decree-Law 54/2018, Art. 10).

More importantly, the new decree-law moves away from the notion that it is necessary to categorise to intervene, rather supporting the idea that all students can achieve a profile of competencies and skills at the end of their compulsory education career, even if they follow different learning paths. Therefore, it views flexible curricular models, systematic monitoring of the effectiveness of the implemented interventions, and an ongoing dialogue between teachers and parents or other caregivers as “the educational responses necessary for each student to acquire a common base of competences, valuing their potential and interests” (Decree-Law 54/2018, Introduction). This approach is similar to the Response to Intervention approach used in the USA in its attempt to “eschew categorisation” (Liasidou 2015, p. 70), which, as discussed by Ferri and Ashby, does not necessarily result in more inclusion. Instead, it often translates in practice to “Tier 1 as the general classroom, Tier 2 as small group instruction, and Tier 3 as one-on-one instruction” (Ferri and Ashby 2017, p. 26).

The new regime also advances a more holistic perspective on the educational process, emphasising that inclusive education is not just the responsibility of special education teachers and other specialised support staff, but rather it must mobilise an interdisciplinary team and, indeed, the school community as a whole. Nevertheless, in order to make schools accountable for all students, the law stipulates the use of Individual Education Plans (IEPs). Even though the responsibility for coordinating the IEP process falls on the mainstream teacher, there are considerable limitations of using IEPs, which often accumulate a number of roles and become bureaucratic instruments rather than educational ones (Millward et al. 2002; Alves 2017). Among the changes introduced to support the education of students with disabilities, the new decree-law creates Learning Support Centres that replace the former Specialised Units. Defined as “dynamic, plural spaces, which assemble both human and material resources” (Decree-Law 54/2018, Introduction), these centres will work with students with disabilities and teachers to support inclusion and promote learning. Reference schools—schools which concentrate specialised resources for teaching low-vision/blind or hard-of-hearing/deaf students—continue to operate.

Overall, the decree-law offers a vague definition of what ‘inclusion’ entails, presented among other general principles, as “the right of all children and pupils to access and participate, fully and effectively, in the same educational contexts” (Decree-Law 54/2018, Art. 3c). If we consider the three pillars of inclusion, this definition omits the notion of success or achievement. Moreover, Anastasiou et al. (2020) point out that this definition conveys a “spirit of sameness” which may indeed put at risk the chances of *all* students with disabilities to get the quality education they are entitled to, and in particular regarding students with learning difficulties. The present policies leave a wide space for the processes of “social, cultural and emotional construction and interpretation” of policy (Maguire et al. 2015, p. 486), which are part of any process of policy enactment. While recognising that inclusion must be a principle of any quality education system, the lack of clarity regarding the processes may be providing the flexibility Priestley and colleagues conclude that is needed to change the “social practices of teaching”, but these changes require “teacher agency” (Priestley et al. 2012, p. 211). However, in a global context marked by “competitive school cultures in neoliberal, marketised, school systems” (Walton 2018), the imprecision regarding the processes of implementing inclusive education may in fact compromise educational success in some schools with lower commitment to the success of all pupils.

Decree-Law 54/2018 was first amended in September 2019, a year after the entry into force of the new legislation. The amendment brought about greater power to parents and caregivers, who are now recognised as “variable members of the multidisciplinary teams” (Law 116/2019, Art. 4a) and entitled to participate in the elaboration and evaluation of technical-pedagogical reports, in addition to the IEPs as the previous decree-law already allowed. On the other hand, schools (through their interdisciplinary teams) are now required to define indicators to assess the efficacy of the measures implemented (Law 116/2019, Art. 5). Additionally, the government took on the responsibility to develop “within 90 days” the statistical indicators for evaluating its inclusive education policy (Law 116/2019, Art. 33.7). As of today, a policy defining this system of evaluation is yet to be adopted. Additionally, the government is required to ensure the necessary means so that education staff in public schools can access free specific training to support inclusion and learning (Law 116/2019, Art. 27). These are examples of the complex process of policy enactment which Maguire et al. (2015, p. 485) have described as being a “jumbled, sometimes ambiguous, messy process that is experienced on the ground by policy actors”.

## Inclusive education in Portugal: What do the numbers say?

More than 10 years since the enactment of Law 3/2008, which strengthened the commitment to inclusive education in Portugal and was reinforced by the Inclusive Education Act of 2018, it is important to monitor these commitments. This section draws on available quantitative data to assess how the three pillars of inclusive education—access, participation, and achievement—are being translated from principle to practice in Portugal.

### Presence or access?

As previously discussed, access is not restricted to physical access to the school premises, but the indicator of presence is one of the easiest to monitor, even if it only provides us limited information with regards to barriers to access, participation, and success. Data on students with “Special Educational Needs” (SEN, the term used by the Office of Statistics on Education and Science—DGEEC) is available from 2009/10 onwards and in little under a decade, there was considerable progress in integrating children with into mainstream schools in Portugal. No data on compulsory education has been released since Law 54/2018 came into force, according to the DGEEC because the indicators are being reviewed in light of the new legal framework. Cross-country data regarding inclusive education in Europe (EASNIE 2017) shows that in the 2016/17 schoolyear, 99.89% of students in basic education (ISCED levels 1 and 2) in Portugal attended mainstream schools, placing Portugal above the European average (98.49%). The same pattern is found in secondary education (ISCED level 3), with 100% of students in mainstream education in Portugal, above the EU average of 97.66%.

The number of students in special education schools decreased by more than a third (37%) since 2010/11 (see Table 1), while the number of students with disabilities in mainstream schools almost doubled (+ 92%). As a result, nearly all students with disabilities in Portugal are currently enrolled in mainstream schools (98.9% in 2017/18)—the majority in public mainstream schools (87.3%), though the number of students with disabilities in private mainstream schools has increased sharply in recent years (+ 413% between 2010/11 and 2017/18).

**Table 1:** Students with special educational needs, per type of school and school year (2010/11 and 2017/18). *Source*: DGEEC (2011, 2018)

	2010/11		2017/18		Variation 2010/11–2017/18
	n	%	n	%	%
Mainstream schools	**45,395**	**96.7**	**87,039**	**98.9**	**+ 92**
Public mainstream schools	43,248	95.3	76,028	87.3	+ 76
Private mainstream schools	2147	4.7	11,011	12.7	+ 413
Private special education schools	**1555**	**3.3**	**984**	**1.1**	**− 37**
Total	**46,950**	**100**	**88,023**	**100**	**+ 87**

The increase in the number of students with disabilities, since 2010/11, was felt in all levels of education (see Table 2). The steepest increase in presence was registered in secondary education (+ 401%), which could be largely due to the extension of compulsory education in Portugal to 12 years, in 2012 through the Decree-Law 176/2012.

**Table 2:** Students with special education needs in mainstream schools, per level of education and school year (2010/11 and 2017/18). *Source*: DGEEC (2011, 2018)

	2010/11		2017/18		Variation 2010/11–2017/18
	n	%	N	%	%
Preschool	2526	5.6	3559	4.1	+ 41
Basic education	39,872	87.8	68,465	78.7	+ 72
Secondary education	2997	6.6	15,015	17.3	+ 401
Total	45,395	100	87,039	100	+ 92

An analysis of gender distribution shows an under-representation of girls identified as having disabilities in mainstream public and private schools: 38%, in comparison with 62% of boys with disabilities. In special education schools this gap is even wider: 72% males vs. 28% females, a 44 percentage-point difference (DGEEC 2018). Pinto and Pinto (2017) suggested that this may be partially due to the social construction of gendered expectations of school achievement and appropriate behaviour. These expectations, they argued, lead to an under-diagnosis of disability among the female student population, which may affect their possibility of receiving proper educational support.

Additionally, data suggests that over half (57%) of the students with complex needs (those with Specific Individual Curricula, Profound and Multiple Disabilities, visual or hearing impairments, or autism) spent less than 40% of the time with their classmates (DGEEC 2018). For 31% of these students, the time spent with the rest of the class was even lower (<20%).

For some of these students, additional support is essential to access learning. Following Decree-Law 3/2008, most Special Education Schools were converted into Resource Centres for Inclusion (CRIs), providing support for students included in mainstream settings, even if a small number of Special Education Schools have continued to provide segregated learning. In 2017, there were 93 CRIs in Portugal (DGE 2017). Specialised support in mainstream schools may be provided by special education teachers, professionals linked to the CRIs, and specialised professionals hired directly by the schools (when no CRI is available nearby). Specialists connected to the CRI or hired by the schools may provide a range of therapeutic supports, including psychological support and speech, occupational, and rehabilitation/physical therapy. In many schools, much of this support is provided individually or to small groups of pupils out of the mainstream classroom (Alves 2015). Despite a sharp increase in the number of students with disabilities between 2010–11 and 2017–18 (+ 92%), the specialised staff to support these students in mainstream schools (CRI and school specialists) increased by only 8% (DGEEC 2011, 2018).

Regarding the type of adaptations provided to students with disabilities in mainstream schools (public and private), data from 2017–18 (DGEEC 2018) shows that the most frequent accommodations were personalized educational support (95.4%) and changes in evaluation methods (89.2%). Almost half of the students with disabilities in mainstream schools had minor curricular adjustments (46.4 %) and 14.4% had more significant curricular adjustments (SIC). This stands in sharp contrast with special education schools, where the percentage of students with SIC is considerably higher—85% (N=839) in 2017–18 (DGEEC 2018).

In 2017–18, 2156 students in mainstream schools received support from a specialized support unit for students with Profound and Multiple Disabilities, blindness, and/or deafness, an increase of 31% since 2010–11 (DGEEC 2011, 2018). There was an even greater increase in the number of students supported by specialized units for autism spectrum disorders: + 73%, from 1221 (2010–11) to 2117 (2017–18). Additionally, there are 32 reference schools providing specific support for blind and visually impaired students (e.g., Braille literacy, orientation and mobility, assistive devices, & daily life and social skills training) (DGE 2018a), and 17 reference school for bilingual education of deaf learners (DGE 2018b). Students can also access assistive technology, through one of the 25 Information and Communication Technology Resource Centres for Special Education—CRTIC (DGE 2020). The budget allocated to these resources in 2018 was 400,000€ (Pinto and Pinto 2019).

While the data presented so far can help us understand the exceptional levels of presence of student with disabilities in mainstream schools, and different types of support available for students with disabilities, they do not allow us to assess students’ overall levels of access or barriers to education. There is no data available regarding the general accessibility of basic and secondary education schools in Portugal. This type of data (e.g., physical accessibility, accessibility of websites, and other indicators) is collected for higher education institutions (DGEEC 2020), but not for compulsory education.

There is also a significant gap in information regarding the outcomes of inclusive education in Portugal. For example, there is no disaggregated data regarding the academic outcomes of students with and without disabilities, either in terms of traditional measures of educational “success” (e.g., success rates, school completion, literacy indicators), or other measures of educational achievement (e.g., acquisition of skills and actionable knowledge, satisfaction), thereby hindering longitudinal or cross-country comparisons of the achievements of Portugal’s inclusive education system. Still, data from the most recent OECD Teaching and Learning International Survey, TALIS (OECD 2018), suggests some challenges which may compromise Portugal’s success. Only little more than a third (39%) of the surveyed teachers, in Portugal, felt prepared to work in an inclusive environment with students with diverse educational needs (OECD 2018), and 27% claimed they would like to receive additional training regarding children and youth with disabilities: 5 percentage points above the OECD average (22%). Also, almost half of the surveyed school principals considered that the quality of education in their school was hindered by a shortage of teachers with competence in teaching students with special educational needs (48%, considerably above the OECD average of 32%).

Overall, the numbers show important progress on the road to inclusive education in Portugal, with almost all students with disabilities currently attending mainstream schools, for the most part public schools, and a considerable increase in the number of students with disabilities in all cycles of learning, particularly in secondary education. Still, the numbers also highlight a number of barriers and challenges. Despite some increase in specialised resources over the past decade, the reinforcement of means is feeble in comparison to the sharp increase in the number of students with disabilities. Moreover, even if they are formally included in mainstream education, students with greater support needs are still spending most of their time segregated from the rest of their class. In addition, it is impossible to know whether decisions regarding placement do consider the best interest of the students. This analysis also shows limitations in data availability regarding the achievements of inclusive education, both in terms of traditional indicators of academic outcomes of students from different groups, and of more nuanced indicators of achievement (e.g., skills and actionable knowledge), thereby hindering national and cross-country comparisons of the achievements of the inclusive education system in Portugal.

## “Achievements” and “needs” of a system: Public opinions of inclusive education

This section will briefly discuss the perspectives presented in the media regarding the national policy changes and inclusive education in Portugal. When trying to map the perspectives of teachers, parents, and students about the development of inclusive education, the paucity of available research data renders it difficult to create a robust picture. Most of the recent data used by the media has been collected and published by teacher unions (Federação Nacional de Educação: FNE, Federação Nacional dos Professores: FENPROF).

The general impression is that there is a sense of achievement and a high level of agreement across the country regarding the principles and values of the new policy. The minutes from a Parliamentary Audition (14.03.2019) reflect this, through praise from the Secretary of State for Education who “showed his appreciation for the consensus at the level of the principles of the policy” (2019, p. 2), as well as members of the opposition who stated “we all agree with regards to the principles” (Assembleia da República 2019, p. 3). The media reported that:

[E]ducational policies in Portugal in the last two decades have led to the inclusion of almost all children and youth with disabilities in mainstream schools. Portugal is a country with beautiful laws, good specialists and professionals who are solidary and able to improvise, so we have reached unprecedented results in special education in Portugal. In 2017/2018, students with special educational needs represented 7% of the public school population. Schools changed to accommodate these students and nothing else was expected. Specialist cluster schools were created for students with specific disabilities: hearing and visual impairments, autism and profound and multiple disabilities. Our situation is unique and inclusion in public schools nowadays covers most children and youth with disabilities. (Soares 2018, p. 2)

This quote suggests not only an increase in the access to mainstream schools but also changes to the educational system to realise the participation of students with disabilities. However, the present policy framework encompasses a wider target population, not only students with disabilities. This perspective is reflected in the media, which also reported that the policy has broadened its scope to all students—not only those with permanent needs—and that the change was praised by parents, teachers, and specialists (Silva 2017). In 2019, another nationwide daily newspaper, *Diário de Notícias*, based on the data collected through the FNE’s questionnaire, reported that many teachers agree with the change of approach in the new policy from “only students with SEN” to “all students” (Reis 2019).

On the other hand, there seems to be some disagreement about not using the concept of Special Education Needs: some believe that this might leave students with SEN forgotten or “left behind”, particularly students with “significant SEN” (Correia 2017). For example, one of the teachers’ unions (FNE) proposed a return to the concept of Special Education Needs, justified by the need to identify differences in order to respond to students with “different issues” (Reis 2019). Others perceived it as a positive step; for example, a parliamentarian (Bebiana Cunha) stated that “school should be, above all, a place of inclusion where each student can find their place and their voice to develop their talents, gifts or potentialities…even though the concept of special needs has finally transformed into inclusive education, based on recognising the right to universal education, equity, inclusion and curriculum flexibility, there is a lot to do based on the difficulties raised by staff and families across the country” (Grupo Parlamentar PAN – Pessoas, Animais, Natureza 2019).

A criticised aspect was the timing and process of change, as the policy was published at the end of the academic year and expected to be implemented at the start of the next year. *Diário de Notícias* reported that Ana Sofia Antunes, Secretary of State for Inclusion, stated that “schools will never be prepared if we don’t pressure them to be. When the previous law was approved (DL3/2008) the schools were also not prepared to do it, but they did. At the time the change was more radical than today. Some did it well, others more or less” (Reis 2019, p. 2). This disparity in application of the policies between schools has also raised concerns about equity between schools (e.g., FNE) (Reis 2019). However, the variation in the enactment of policy is well documented in the literature: policies are always enacted in specific ways in each context, depending on “situated contexts (e.g., local school histories and intakes), professional cultures (e.g., values, teacher commitments and experiences and “policy management” in schools), material contexts (e.g., staffing, budgets, buildings, technology, infrastructure), external contexts (e.g., degree and quality of LA support; pressures and expectations from broader policy context, such as […] league table positions, legal requirements and responsibilities)” (Ball et al. 2012, p. 21).

Media reports also reveal disagreement between stakeholders and potential challenges with implementing the new policy (Silva 2017), particularly with regards to a lack of resources and a lack of clarity. *O Público* reported on three stakeholders’ perspectives. Ana Simões, a member of FENPROF, stated that “The ‘issue’ is how the policy vision will be applied in practice. For a true inclusion we need resources, and that cannot be achieved when we expect to use only resources already existing at school level”. David Rodrigues, representing a national organisation of special education teachers (Pró-Inclusão), similarly noted that without resources, the implementation of the policy would be uncertain. And finally, Luisa Beltrão, from an organisation of parents of children and young people with disabilities (Pais em Rede), stated that “the proposal makes sense, but we need to be realistic. The policy hasn’t been adopted in most schools. There isn’t a single school in Portugal that we consider inclusive. So having a policy which is even more demanding applied to a system which wasn’t able to do basic things and continues to exclude these children is problematic” (Silva 2017).

The lack of human resources seems to be one of the key issues, with some parents claiming that their children were left without support. For example, the mother of an autistic child stated that “these students’ needs didn’t disappear, but they were put in stand-by” (Viana 2019a). This could be linked to the process of schools having to file a request with the Ministry of Education when additional resources are required, which might delay the response even when the resources are made available. Research from Scotland reported competing discourses between children’s desires (e.g., to be seated beside their friends) and teacher’s perspectives (need for extra support), emphasising the need to engage with student voices when assessing a system’s achievements and needs (Allan 2008).

Also linked to resources are decisions regarding class sizes. To justify a decrease in class size, the policy requires students with particular needs to spend at least 60% of their time in the mainstream classroom. This was interpreted by the media as pressure to ignore “mainstream” students’ individual needs, especially since only 28% (of 12550) of students with “severe limitations” (who often need specialised support outside the classroom) were reported to spend at least 60% of the time in their mainstream classroom (Viana 2018). While smaller class sizes might benefit all students and teachers, students with complex needs represent less than 0.8% of the entire school population, and if they spend most of their time outside the mainstream classroom, there is little justification for reducing the number of students in their mainstream classes.

Considering the reported “lack of clarity”, the new legislation was followed by a “manual for implementation”, but both have been criticised for being vague. FNE noted, for instance, that “the interventions proposed are subject to multiple interpretations and forms of intervention according to the interpretation” (Reis 2019). The media reported uncertainty in the implementation as to which students must have a technical-pedagogical report, which provides access to specialised support and accommodation: speech and language therapists and adaptations in exams, such as extra time (Viana 2019a). At the previously mentioned Parliamentary Audition (14.03.2019), parliamentarian Diana Ferreira stressed the value of special education teachers, who she said “should not be consultants and should coordinate the multidisciplinary teams” (Assembleia da República 2019).

According to the results of a questionnaire distributed by FENPROF (answered by 1192 of 110,000 teachers and 92 of 801 school leadership teams) “headteachers were happy, and teachers unhappy” regarding the enactment of the new policy (Viana 2019b). Perhaps this more positive approach from school leadership teams could be related to closer communication with the Ministry of Education, as it has been stated that “the Ministry of Education meets headteachers every 3 months since the new policy was published in order to clarify concepts in the first year” (Viana 2019b). The attempt to keep communication channels open seems to be a priority in this process of policy change: for example, a parliamentary public hearing was organised 5 months after the new policy came into effect in schools, featuring over 200 participants. Researcher Marisa Carvalho (2019) reported in the media that based on a study with 800 teachers, the main challenges were a lack of human and material resources, lack of teacher education, and organisational structures unfit for the purpose. Conversely, Carvalho (2019) noted that school leadership teams, when asked about the role of school leadership in inclusion, suggested that the focus should be on finding and developing solutions for their school in a collaborative, participative, and proactive way, rather than looking for the answer in policy or waiting for central guidelines.

## Discussion and conclusions

In this article we examined the evidence of and challenges to the development of inclusive education in Portugal. We started by briefly outlining current conceptualisations of inclusive education, then considered the role of curriculum, pedagogy, and assessment in the development of inclusive policies and practices. We then discussed the endeavour of ensuring inclusive and equitable quality education for all in the Portuguese context. In this discussion section, we will consider both the present policy framework and the existing evidence presented earlier, to evaluate Portuguese efforts toward inclusive education. To do so, we take into account the three key aspects of inclusion—access, participation, and achievement of all students.

It is fair to say that the Portuguese system has continuously improved at giving all learners physical access to education during the 12 years of compulsory schooling, with school dropout rates steadily decreasing from 50% in 1992 to 10% in 2019 (Instituto Nacional de estatística and PORDATA 2020). In addition, the presence of students with disabilities in mainstream schools is close to 100%. However, the inclusion of students in education is not realised by simply “placing students with disabilities within mainstream classes without accompanying structural changes to, for example, organisation, curriculum and teaching and learning strategies” (UN 2016, p. 4). The present policy framework, not only through DL54/2018, but also through the Student Profile at the end of compulsory schooling (Ministério da Educação 2017) and the Decree-Law 55/2018, attempts to align curriculum and assessment to make them applicable to all students, rather than to the convenient majority.

In this regard, Norwich (2010, p. 132) distinguishes between different aspects of the curriculum:

1. general principles and aims for a school curriculum (principles)
2. areas of worthwhile learning (whether structured in terms of subjects or not) with their goals and general objectives (programme areas)
3. more specific programmes of study with their objectives (specific programmes)
4. pedagogic or teaching practices (teaching).

Using Norwich’s (2010, p. 132) categorisation, the endorsement of the Universal Design for Learning principles (CAST 2018) demonstrates an attempt to work with inclusive “general principles and aims”, “areas of worthwhile learning”, and “pedagogic or teaching practices”. This reform also couples inclusive general goals and areas of learning with principles of curricular flexibility at the level of “more specific programmes of study with their objectives” (Norwich 2010, p. 132), in an attempt to enact the necessary “process of systemic reform embodying changes and modifications in content, teaching methods, approaches, structures and strategies in education to overcome barriers” (UN 2016, p. 4).

While the new policy has tried to align the “message systems” (Lingard and Mills 2007) of curriculum and pedagogy, there is still some conflict at the level of assessment. As part of this reform, “levels of inclusivity” have been reported to be part of school assessment, and both schools and the Ministry of Education are expected to develop indicators to monitor levels of inclusivity and success in implementing the current policy framework. The development of inclusive education systems requires a critical reflection on the meaning of “success”, however. If success is conceptualised in non-inclusive ways—that is, if we continue to evaluate success merely through the number of students reaching certain grades in a limited range of subjects—the schools’ priorities might not lie in creating inclusive schools. Documents such as the Index for Inclusion (Booth and Ainscow 2011) and the Guide for Ensuring Inclusion and Equity in Education (UNESCO 2017) provide useful tools for developing monitoring systems that take into account the voices of stakeholders involved (both teachers and pupils) from a perspective of action research.

Additionally, the current policy framework proposes abandoning both the “special educational needs” category and the broader need to categorise a student’s needs before intervening. The issue of “identifying” or labelling students is a well-researched area with conflicting views (Biklen et al. 1997; Ho 2004; Corbett 1994; Lauchlan and Boyle 2007). It is also one of the dilemmas of difference (Minow 1990), when applied to inclusive education (Norwich 2008, 2009), i.e., a situation where “there is a choice between alternatives when neither is favourable” (Norwich 2010, p. 117). Eliminating the conceptual use of SENs might not have the desired impact on practice, and creating a new label, “special health needs”, does not seem to align with the policy discourse of removing the need to categorise to intervene. On the other hand, removing the need to categorise to intervene should be an important step towards a more inclusive system, in which the responses depend on the need for additional support rather than on a medical diagnosis of “permanent health conditions”, as in the previous legislative framework.

Nevertheless, effecting this vision will require a significant investment in teachers, to support their agency in the process of enacting the policy and enacting a paradigm shift (Alves 2020). Rouse (2008) proposed that bypassing labels to access support, as proposed in the current legislation, requires practitioners to develop knowledge about inclusive pedagogy (Black-Hawkins and Florian 2012). Moreover, practitioners must “believe that they have the capacity to make a difference to children’s lives” (Rouse 2008, p. 14) and that developing inclusive education is “not only a task for specialists working with special education needs students” (Rouse 2008, p. 14). Instead, it requires mainstream teachers to collaborate with others (including students, colleagues, other professionals, parents) with the aim of removing barriers to inclusion, and to use evidence to improve practice (Messiou 2019). These aspects will only be achieved through a reorganisation of schools’ time and spaces, to allow for collaborative approaches, as well as through initial teacher education and continuous professional development, which should involve opportunities to research barriers to inclusion and engage with pupil voices.

While foreseen in the new legislation, so far these goals have been difficult to achieve. This brings our attention back to the issue of policy enactment, and how “policy practices are specific and contextualised. They are framed by the ethos and history of each school and by the positioning and personalities of the key policy actors involved” (Braun et al. 2010, p. 558). In the Portuguese context there seems to be some resistance from a considerable number of former Special Education teachers regarding their new role, as well as dissatisfaction among teachers in general with the new requirements placed on them, mostly due to lack of resources and training. The change in paradigm for supporting students with additional needs has also been perceived by some as a disinvestment in education and a devaluation of Special Education Teachers, as expressed by parliamentarian Diana Ferreira. However, the variation between schools in policy enactment and in developing context-sensitive and inclusive responses is clear, acknowledged, and even supported by the Ministry of Education, through the existence of “lighthouse” schools. Similar findings were reported earlier (Darling-Hammond 2017, p. 305), albeit with a different focus.

Emergency responses to the current COVID-19 pandemic forced a move to distance education. This has allowed us, on one hand, to realise the multiple roles of school, including a welfare aspect: schools are important institutions for students from disadvantaged backgrounds (e.g., free school meals). The move to distance education has also opened new channels of communication with learners (e.g., #estudoemcasa—a study-at-home television programme developed by the Ministry of Education). However, this situation has also revealed some accessibility challenges: certain students were “left behind” or excluded due to lack technology access or activities that are not appropriate for students with certain disabilities. Perhaps more to the point, it has shown that relationships are at the core of teaching, underscoring the challenge of creating and maintaining online relationships between teachers and students, especially students with severe and complex needs. Overall, the present situation has brought to the surface the challenge of reducing barriers to and reducing inequalities in education. It is also a strong illustration of the fragility of inclusive education and the need for a secure commitment to equity, dedicated to the right to education of all children. In closing, the main justification for developing inclusive education systems is to realise the basic human right to education of all children and young people, which will only be achieved through a continuing commitment to promoting the access, participation, and success of all students.
